# Finite-element modelling of mechanobiological factors influencing sesamoid tissue morphology in the patellar tendon of an ostrich

**DOI:** 10.1098/rsos.170133

**Published:** 2017-06-07

**Authors:** Kyle P. Chadwick, Sandra J. Shefelbine, Andrew A. Pitsillides, John R. Hutchinson

**Affiliations:** 1 Structure and Motion Laboratory, Department of Comparative Biomedical Sciences, The Royal Veterinary College, Hatfield, UK; 2 Department of Mechanical and Industrial Engineering, Northeastern University, Boston, MA, USA; 3 Skeletal Biology Group, Department of Comparative Biomedical Sciences, The Royal Veterinary College, London, UK

**Keywords:** sesamoid, biophysical stimuli, tissue differentiation, mechanobiology

## Abstract

The appearance and shape of sesamoid bones within a tendon or ligament wrapping around a joint are understood to be influenced by both genetic and epigenetic factors. Ostriches (*Struthio camelus*) possess two sesamoid patellae (kneecaps), one of which (the distal patella) is unique to their lineage, making them a good model for investigating sesamoid tissue development and evolution. Here we used finite-element modelling to test the hypothesis that specific mechanical cues in the ostrich patellar tendon favour the formation of multiple patellae. Using three-dimensional models that allow application of loading conditions in which all muscles, or only distal or only proximal muscles to be activated, we found that there were multiple regions within the tendon where transformation from soft tissue to fibrocartilage was favourable and therefore a potential for multiple patellae based solely upon mechanical stimuli. While more studies are needed to better understand universal mechanobiological principles as well as full developmental processes, our findings suggest that a tissue differentiation algorithm using shear strain and compressive strain as inputs may be a roughly effective predictor of the tissue differentiation required for sesamoid development.

## 1. Introduction

Sesamoid bones, or bones that are found within a tendon or ligament near a joint, have broadly been neglected in a comparative and developmental context [1,2]. Tsai & Holliday [3] described an unusual sesamoid structure, made of fibrocartilaginous tissue, in the jaw joint of Crocodylia, suggesting that sesamoids need not only be ossified structures. Samuels *et al*. [4] reviewed and updated information on the evolution and development of patellar sesamoid bones in mammals, finding about five independent origins of a bony patella in Mammaliaformes and multiple losses (and possibly regains) in marsupials and other lineages. Regnault *et al*. [5] discovered that the closest living relative of lizards and snakes (Squamata), the tuatara (*Sphenodon*), has a bony patella, raising the question of whether this trait is ancestral for both of these clades. While the appearance and shape of sesamoids are understood to be influenced by both genetic and epigenetic factors, the specific mechanical cues underlying their transformations are unknown [6].

**Figure 1.**
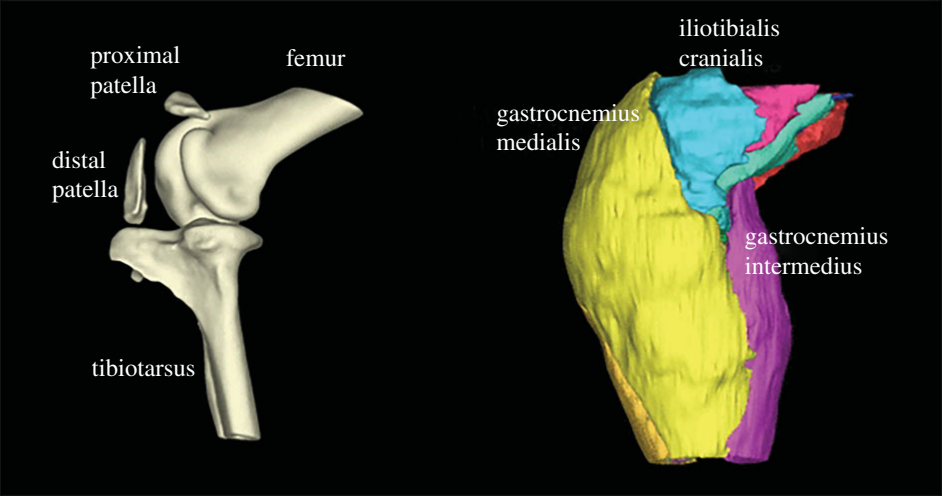
Adult ostrich knee skeletal anatomy reconstructed from computerized tomography (left) and muscular anatomy reconstructed from magnetic resonance imaging (right), medial view; with major structures labelled.

Ostriches (*Struthio camelus* Linnaeus 1758), the largest living birds, have two patellae (kneecaps; tibial sesamoids) located proximally and distally within the patellar tendon [7]. The rest of ratite birds split from ostriches approximately 80 million years ago [8,9]. As shown in figure 1, the morphology observed in the knee joint of ostriches is unique in many ways [10], with the rest of ratites possessing a single small flake of bone proximally, no patellae at all, or ambiguous morphology [11]. The patellae of ostriches are embedded in a thick fascial layer that continues from the proximal patella to the proximal end of the distal patella. Uniarticular, femorotibial muscle–tendon units insert onto the proximal patella. Other parts of the femorotibial complex, and the biarticular hip flexor/knee extensor M. iliotibialis cranialis, insert onto the distal patella. Furthermore, the distal patella is the partial origin for distal limb musculature including the lateral and medial gastrocnemius as well as M. fibularis longus (figure 1). Chadwick *et al*. [10] hypothesized that this anatomy of muscles, tendons and sesamoids would result in complex compressive and tensile stresses on the patellae as well as shear loading in the tissues surrounding the femorotibial joint.

No other ratites (or, indeed, other birds yet described; to our knowledge) have a distal patellar sesamoid. Curiously, the distal patella comes very close to having a direct articulation with the tibial crest (crista cnemialis cranialis) of the tibiotarsus, with only an abbreviated strand of connective tissue interposed between them. In contrast, the single small flake of bone found in some ratites seems to be the ancestral form of the patella for crown group birds plus some close relatives (the clade Ornithurae), but as the latter study described, many lineages of birds deviated from this form. Such deviations, as in ostriches, raise interesting questions about biomechanical consequences of changes in sesamoid bones (e.g. for muscle and joint function, or more broadly for locomotor behaviour) as well as about developmental (e.g. mechanobiological) factors involved in evolutionary transformations of form and function. Following a detailed anatomical investigation [10], we now aim to investigate, using computer simulations, whether this difference in ostrich patellar form and development is spatially linked to any specific mechanobiological factors.

**Figure 2.**
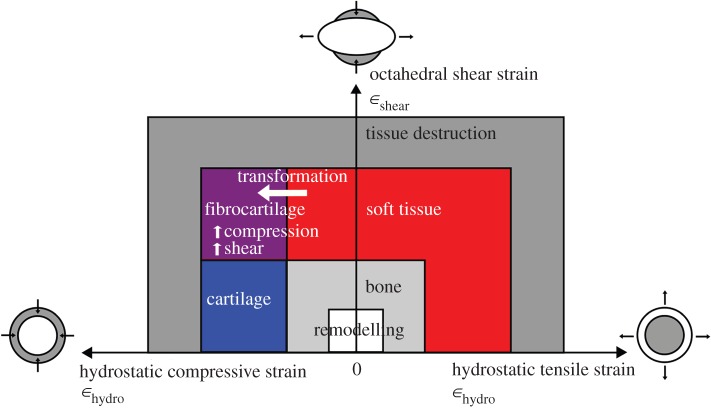
Tissue differentiation diagram based on Shefelbine *et al*. [12]. The horizontal axis represents positive (tensile) to negative (compressive) hydrostatic strain. The vertical axis represents low to high octahedral shear strain. This study models the transformation from soft tissue (red zone) to fibrocartilage (purple zone) based on the criterion of high compression and high shear.

Mechanical factors are known to influence many skeletal processes, including epiphyseal development, long bone growth, sesamoid cartilage ossification and fracture healing, and all have been subjected to mechanobiological finite-element modelling. This modelling of tissue differentiation (see figure 2) is based either upon linearly elastic or poroelastic material properties. The latter takes into account fluid flow through the tissue [13–16] and because this largely occurs on a time scale longer than that of most physiological loading, a linearly elastic model is thought to be nearly equivalent over short time intervals [17]. We therefore chose to apply a quasi-static model (i.e. infinitesimal time interval) and to use linearly elastic modelling in this tissue differentiation framework to explore, herein, the spatial relationships between specific mechanobiological factors in loaded ostrich patellar tendon and the singular phenomenon of multiple patellae development in ostriches.

In order to identify mechanical environments that favour different tissue transformations, linear elastic studies have used two parameters to map stress/strain states onto two-dimensional diagrams that define tissue differentiation [12,14,18–21]. Figure 2 shows a diagram which represents the general insights of these previous diagrams, independent of absolute boundaries; for further details, see the publications cited above.

Here, we model the mechanobiology of the ostrich patellar tendon in order to examine its potential for tissue transformation. Our model is three-dimensional and thus captures the unusual and complex loading that is expected to prevail in this region, providing an advance in mechanobiological analysis. We hypothesize that both the proximal and distal patellae have developed as a result of a mechanical environment which favours multiple centres of ossification. Models of an ostrich's patellar tendon with digitally removed patellae were constructed in order to test this hypothesis.

## 2. Methods

In order to clarify the mechanobiological factors underlying why the two ostrich knee sesamoids develop, a representative adult ostrich's (71.3 kg, skeletally mature male) patellar tendon was modelled in finite-element analysis software (Abaqus, Dassault Systèmes Simulia Corp., Boston, MA) under simulated *in vivo* loading conditions. The patellae were digitally removed, and the tendon was modelled as a continuous and homogeneous tissue. This allowed for the modelling of both (approximately) the early evolutionary and early ontogenetic states of the patellae in ostriches. The ostrich cadaver specimen used was from a prior study [10] and had been acquired from a local farm after being euthanized for reasons unrelated to this study.

Previous studies of the mechanobiology of a tendon wrapping around a joint have exclusively been two-dimensional, with idealized geometry [6,13,19], or representative of embryonic development before fibrous tendon formation [16]. The ostrich knee is also known to have substantial three-dimensional components [22,23]. Therefore, three-dimensional models were employed in this study, with geometry taken directly from a scanned specimen (see below). Many parameters define each model, and these parameters were taken from magnetic resonance imaging (MRI)/computerized tomography (CT) scans, dissections, and past modelling studies, along with some commonly used assumptions in the literature, as follows.

### 2.1. Bone and tendon meshes

The bone and tendon shapes used in the models came from segmented CT and MRI scans (in Mimics software; Materialize, Inc., Leuven, Belgium) that we previously described [10]. The volumes used to model the femur and tibiotarsus were created by first exporting the relevant three-dimensional objects from Mimics to 3-matic software (v. 9.0, Materialise, Inc., Leuven, Belgium). A split in the patellar tendon, separating a deep layer and superficial layer, was observed during anatomical dissections [10]. In order to more realistically model this split in the tendon, a Boolean operation was performed in 3ds Max software (v. 2016, San Rafael, California, USA) using a volume representing the small gap between the tendon parts. In 3-matic, the tendon was converted into a tetrahedral volume mesh formed of 37 767 elements while the femur and tibiotarsus were converted into triangular surface meshes of 12 848 and 22 548 elements, respectively. Results obtained using a more refined tendon mesh (5 937 832 tetrahedral elements) demonstrated similar patterns observed with the primary model mesh.

### 2.2. Muscles and loading

The attachment sites of all muscles were determined primarily through dissections, with segmented volumes [10] supporting these observations. Muscle force magnitudes were taken from a previously generated forward dynamic simulation [24] conducted using a musculoskeletal model [23] of an ostrich pelvic limb. That simulation's subject had a body mass of 78.7 kg but given the modest (10% larger) differences in size and the exploratory nature of this study, the force magnitudes taken from the simulation were not scaled down to match our representative subject's. The experimental data used in the simulation were re-used from a prior study [22] (see also [24]). The kinematics and kinetics for this simulation were taken from slow running (3.46 m s^−1^, duty factor: 0.40, peak vertical ground reaction force: 1897 N (i.e. 2.7 times body weight)) to match the position of the modelled joint. The directions of all muscle vectors were approximated from segmented muscles' volumes and muscle origin/insertion sites [23] (table 1).

**Table 1.** Forces used in the model, taken from *R*_CMCC_ simulation in Rankin *et al*. [24].

muscle group	force (N)	loading group
M. iliotibialis cranialis	66	proximal
Mm. femorotibialis intermedius, femorotibialis lateralis pars proximalis, femorotibialis lateralis pars distalis	1127	proximal
M. gastrocnemius lateralis	501	distal
M. gastrocnemius medialis	547	distal
M. fibularis longus	677	distal

In order to better visualize the effects of different muscle groups, models were created for three different loading conditions: all the muscles activated (most realistic condition; table 1), only muscles proximal to the tendon activated (i.e. thigh muscles such as M. femorotibialis), and muscles distal to the tendon activated (i.e. shank muscles such as M. gastrocnemius). With these three loading conditions represented, we could observe the sensitivity of the results to a spectrum of loading conditions and the direct effects of each muscle group. These conditions enabled us to examine the diversity of forces that might influence sesamoid formation. However, for simplicity only one instant (one posture near peak loading during slow running) was investigated here; data on faster running were not available, and the best anatomical imaging data (from [10]; MRI and CT scans) could only be obtained for the limb orientation used here. Nonetheless, the open nature of the data in our study means that future studies could use these data to examine other morphologies, loading conditions, postures or behaviours.

### 2.3. Boundary conditions and interactions

All the parts in the model were constrained to closely approximate *in vivo* attachments and interactions. During proximal muscle activation simulations, in order to fully constrain the model during each loading condition, the distal end of the tendon was fixed and during distal muscle activation simulations, the proximal end of the tendon was fixed. Surface-to-surface contact was defined between the tendon and rigid femur, and self-contact was defined between the two inner surfaces where the patellar tendon splits. These contact boundary conditions allowed the two surfaces to touch, slide against each other, and transfer stress without intersecting.

### 2.4. Material properties

**Figure 3.**
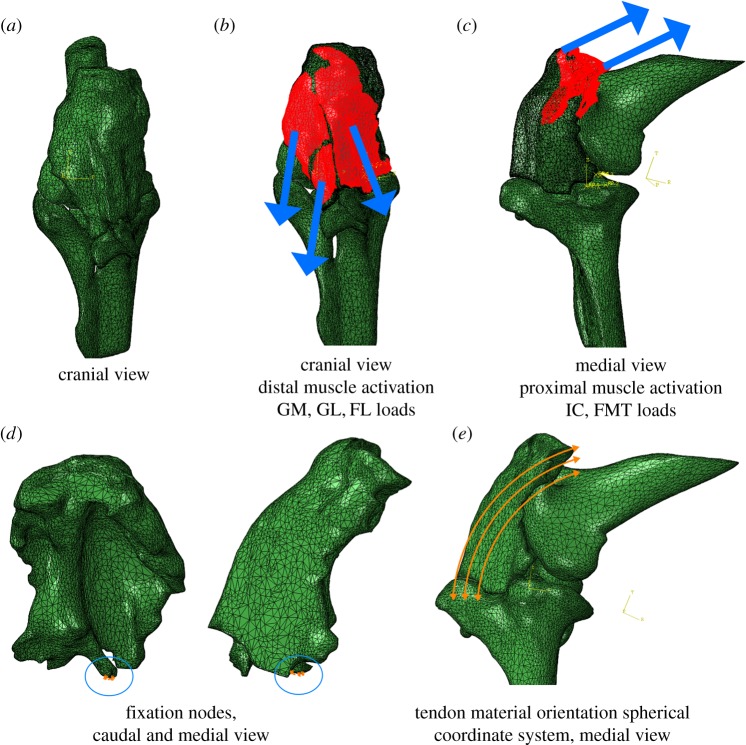
Finite-element model boundary conditions. (*a*) Cranial view of meshed model assembly, (*b*) cranial view of distal loads, (*c*) medial view of proximal loads, (*d*) caudal and medial view of fixation nodes, (*e*) medial view of the spherical coordinate system used for the tendon's material orientation.

**Figure 4.**
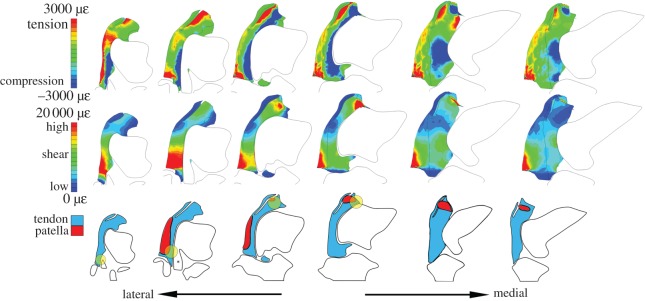
Results from proximal loading model. Compare with figure 3*e* for morphological context. Top row: Tensile (red) and compressive (blue) hydrostatic strain visualized in six section cuts of the tendon. Middle row: High (red) and low (blue) octahedral shear strain visualized in six section cuts of the tendon. Strain gradients are visualized nonlinearly by maximum and minimum values (shown on legend), to prevent saturation. Bottom row: The anatomy observed at each of the six section cuts, including patellae. Yellow circles indicate regions where compressive hydrostatic strain and high octahedral shear strain both occur. *Origin of the M. tibialis cranialis tendon.

The patellar tendon was assumed to be a linearly elastic, orthotropic material. There is a range of material properties in the literature for the hindlimb tendons of birds and other species. The reported longitudinal elastic modulus ranges between about 216 MPa and 1.97 GPa [25,26]. Some of this variation is due to mineralization of the tendons in some smaller bird species, but ostriches show no such mineralization (e.g. [27–29]). Therefore, the higher-end values within that variation would not be realistic assumptions for ostriches. Regardless, because of this variation, we used values similar to those from previous modelling studies to facilitate more direct comparisons. Thus, longitudinal (along-fibre) and lateral elastic moduli were assumed to be 800 MPa and 8 MPa, respectively; Poisson's ratio was 0.497, and the shear modulus was 2.7 MPa [6,13,19]. The femur (including the M. tibialis cranialis tendon origin; figure 4) and tibiotarsus were modelled as rigid bodies. The local coordinate system defining the longitudinal and lateral fibre directions of the tendon was approximated using a spherical coordinate system to simulate tendon fibres three-dimensionally wrapping around the femoral condyles (figure 3).

### 2.5. Mechanobiological stimuli calculations

Additional field outputs needed to be computed after the simulation in order calculate the variables of interest. The hydrostatic strain and octahedral shear strain used in the tissue differentiation diagram were calculated through the use of the first and second strain invariants (equations (2.1) and (2.2)).

2.1 $$I_{1}=\varepsilon_{11}+\varepsilon_{22}+\varepsilon_{33}$$

and

2.2 $$I_{2}=\varepsilon_{11}\varepsilon_{22}+\varepsilon_{22}\varepsilon_{33}+\varepsilon_{11}\varepsilon_{33}-\varepsilon_{12}^{2}-\varepsilon_{23}^{2}-\varepsilon_{13}^{2}.$$

These invariants were used to calculate the hydrostatic strain and octahedral shear strain (equations (2.3) and (2.4)).

2.3 $$\varepsilon_{\mathrm{hyd}}=\frac{1}{3}I_{1}$$

and

2.4 $$\varepsilon_{\mathrm{shear}}=\frac{1}{3}\sqrt{2I_{1}^{2}-6I_{2}}.$$

## 3. Results

We hypothesized that a mechanical environment favouring multiple centres of ossification underpins the development of paired proximal and distal patellae in an ostrich. Through the three loading models, we were able to estimate the influence of each muscle group on the strain within the tendon. Generally, we found that compressive strain occurred where the tendon was being pushed against the femur, tensile strain occurred in the superficial regions where it wrapped around the femur, and shear predominated adjacent to boundary conditions fixing a surface.

### 3.1. Proximal loading model

There were two unique regions of high compression within the proximally loaded tendon (figure 4): located distally and proximally, along the tendon–femur contact surface.

There were also two regions of high shear within the proximally loaded tendon (figure 4). The most pronounced was located distally and mostly superficially, primarily lateral to the M. tibialis cranialis tendon. The second, less pronounced region of high shear was located proximally, just above the lateral femoral condyle.

There were two regions where high compression and high shear coincided within the proximally loaded tendon, consistent with a greater potential for fibrocartilage transformation at these two locations. The region of greatest compression and shear was located around the contact with the M. tibialis cranialis origin and the other region was along the proximal edge just above the lateral femoral condyle.

### 3.2. Distal loading model

**Figure 5.**
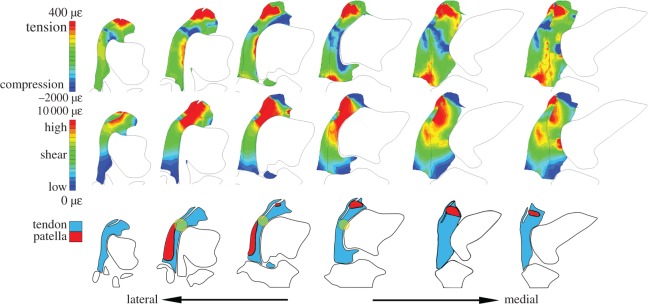
Results from distal loading model, in the same format as figure 4.

There were approximately three unique regions of high compression within the distally loaded tendon (figure 5). The region of greatest compression was located around the interface of the superficial and deep tendon layers, extending to the superficial surface as well as the femoral contact surface. Another region of large compression was located proximally along the femoral contact surface, above the lateral femoral condyle. The final region experiencing considerable compression was located along the tendon's contact surface with the femur, but below the largest prominence of the lateral femoral condyle and medial to the M. tibialis cranialis origin.

There was one primary region of high shear within the distally loaded tendon, which was located above the lateral femoral condyle, extending from the femoral contact surface through the superficial tendon layer.

There was one clear location in the distally loaded tendon in which high compression and high shear coincided, indicating greater potential for fibrocartilage transformation. This was located around the interface of the superficial and deep tendon layers close to the femoral contact surface.

### 3.3. Full loading model

**Figure 6.**
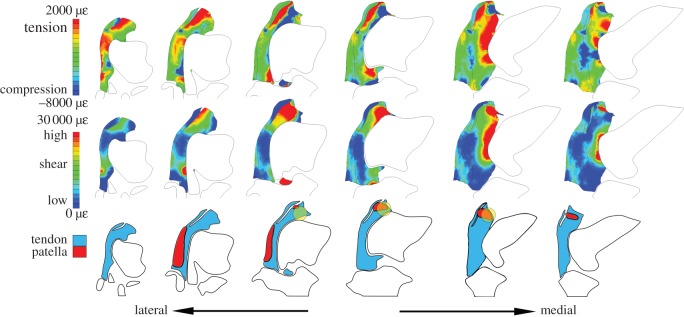
Results from full loading model, in the same format as figure 4.

There were three unique regions of high compression within the fully loaded tendon (with both proximal and distal tensile forces; figure 6). The largest was located proximally along the femoral contact surface, above the lateral femoral condyle. The second largest was located along the tendon's contact surface with the femur, but below the largest prominence of the lateral femoral condyle and primarily medially and laterally to the M. tibialis cranialis tendon's origin. Finally, there was a smaller amount of compression located at the union of the superficial and deep tendon layers.

There were two prominent regions of high shear within the fully loaded tendon. The largest region was located proximally in the deep tendon layer, above the lateral femoral condyle, extending from the femoral contact surface to the superficial tendon layer contact surface. A smaller region of high shear was located along the femoral contact surface, medial to the lateral femoral condyle.

There was one distinct region of the fully loaded tendon in which high compression and high shear coincided, indicating greater potential for fibrocartilage transformation. This was the proximal region along the femoral contact surface and above the lateral femoral condyle.

Based on the results from all three models, we infer that strains favouring tissue transformation into fibrocartilage occur in multiple regions of the ostrich patellar tendon.

## 4. Discussion

The models presented herein indicate, foremost, that there is no single definitive region favouring tissue transformation. Instead, the models indicate that there are multiple, likely two or three such locations in the loaded patellar tendon model. Our data show that compressive strains prevailed (as expected) at the interface between the patellar tendon and femur, while the superficial region of the patellar tendon experienced mainly tensile strains, and the boundary conditions of our model's surfaces resulted in shear strains. These findings are broadly consistent with the predictions made previously by Chadwick *et al*. [10] based purely upon the complex anatomy of the knee joint region in ostriches. Indeed, our finite-element analysis reinforces, to a general, qualitative degree, the predictions of biomechanical loading based upon anatomical form and using only biomechanical principles. The major finding of our analysis, in terms of tissue differentiation theory (figure 2), is that high compressive and shear strains—which would favour a localized enrichment of tissue with fibrocartilaginous composition as well as its ultimate ossification—do roughly match the position of the proximal patella in particular, but this match is not perfect. Additionally, the results of our analysis do not correlate well with the possible emergence of the distal patella as a result of its mechanical environment, which is a curious finding that we explore below. A precise match of our results is not expected, however, given the simple, static nature of our model, the emphasis on one instant in locomotion where moderately large loads occur in one joint posture, and the focus on adult morphology rather than actual ontogenetic transformation. Nevertheless, our results remain relatively robust under diverse loading conditions and are strengthened by the three-dimensional nature of the biomechanical models. These results suggest that unique features in the ostrich patellar tendon morphology and local loading environment may support the development of two patellae, which is consistent with our hypothesis that a mechanical environment favouring multiple centres of ossification is correlated with the development of paired proximal and distal patellae in the ostrich.

**Figure 7.**
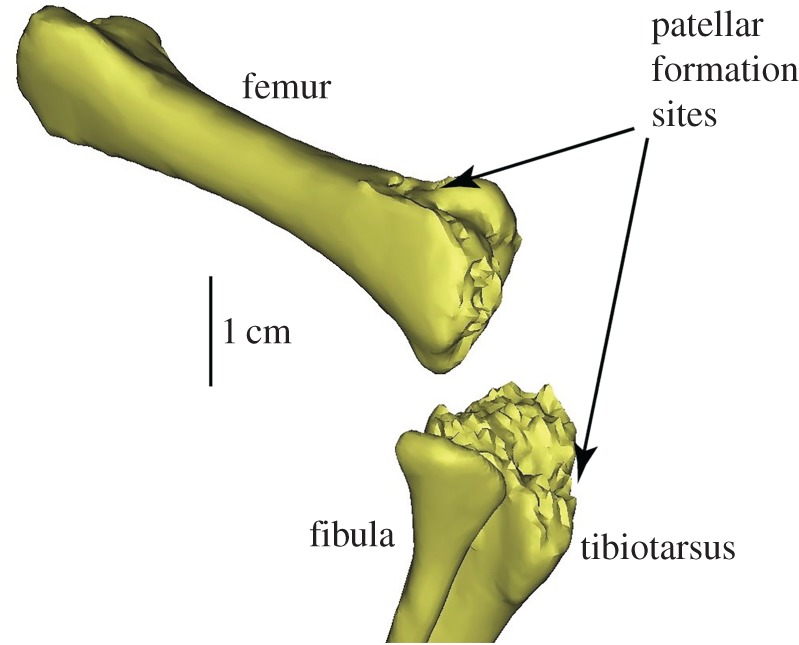
Newly hatched ostrich chick knee (right limb in oblique craniolateral view) reconstructed from CT scan, showing an underdeveloped proximal tibial epiphysis and an absence of patellae (cf. figure 1).

It is likely that ostriches develop their patellae late in their ontogeny, similar to humans. Wagner [30] found that the onset of patellar ossification in ostriches occurred between six months and one year of age. This corroborates with our CT scan of a newly hatched ostrich chick (J.R.H., uncatalogued RVC specimen) that shows no sign of ossified patellae (though a cartilaginous precursor could exist without being seen in CT) and an underdeveloped proximal tibial epiphysis (figure 7). Our further examinations of 10 other specimens of similar age supported this morphology as typical of very young ostriches. More research on sesamoid ontogeny in ostriches and other birds is needed, but if this inference is correct, then modelling an ostrich tendon without patellae is likely also akin to modelling a juvenile (post-hatching) ostrich before patellar development occurs; we acknowledge that size and shape, locomotor dynamics, and tissue properties are likely to differ, and thus our modelling procedure is only a rough approximation.

It is also strongly suspected that the two patellae in the ostrich evolved at different times in ostriches' evolutionary history, with the proximal patella being ancestral for extant birds including ostriches and thus 100+ million years old, whereas the distal patella evolved sometime after ostriches diverged from other birds approximately 80 million years ago [11]. This unusual evolutionary history further justifies the diverse loading conditions (i.e. proximal, distal and full loading models) we used in our study. As it is younger in an evolutionary sense, the distal patella might not only have evolved along a different developmental pathway or even mechanism, but might also exhibit a different sensitivity to its mechanical environment than the proximal patella. It is, regardless, unclear if any avian patellae are more ‘genetically assimilated’ than in mammals or lepidosaurs [2,11]. Illumination of this question would require more studies of embryonic/postnatal ontogeny, ideally across multiple species and loading conditions.

This study provides a pilot framework that future studies can build upon. Without making the model iterative in its material properties based on a feedback loop, similar to Shefelbine *et al*. [12], we are unable to draw any conclusions based on the actual locations of favourable tissue strains within the tendon and their relation to the observed patella locations in the adult ostrich. Also, this type of tissue differentiation analysis has thus far only been applied to fracture healing; however, the mechanisms by which fracture healing develops new bone may differ from those of epiphyseal development, long bone development, and sesamoid ossification.

Herein, we have assumed that the patellar sesamoids form through a pathway (following tissue differentiation theory as in figure 2) involving a fibro-cartilaginous transitional phase. The assumed remaining pathway to endochondral ossification (not included in this study) would be that an increased modulus of fibrocartilage (relative to fibrous soft tissue; e.g. dense tendon) reduces compressive and shear stresses and creates a mechanical environment that ultimately favours ossification. This idea of a cartilaginous transition phase is a commonly held notion, and validated in other species, including some birds, which possess cartilaginous patellae during development [31–33]. The ontogenetic development of ostrich patellae has not yet been well documented, so we cannot be sure that a cartilaginous phase exists although it would be somewhat surprising if there was not one. If ostrich patellae do not form through endochondral ossification, they may form through fully metaplastic ossification, which may follow similar mechanobiologically driven processes but without the cartilaginous tissue intermediary. Considering that patellae are known to have evolved at least three times in tetrapod vertebrates [1,4,5,11,34], it is plausible that one or more of these evolutionary events may involve different routes of differentiation to ossified bone.

Previous studies performed on sesamoid bone development have thus far been limited to two-dimensional models with idealized geometry [6,13,19] or embryonic development before fibrous tendon formation [16]. Our study provides the first analysis of developmental stimuli on sesamoid bone formation in fibrous tendon in three-dimensional models. Therefore, this study could act as an example to be extended to more sesamoid bones (or unossified patellae; e.g. [11]), including when they occur as a single patella, when they articulate directly with bone, and in other independent evolutions of the patellae including those in Mammalia and Squamata [1,5,11,34]. Further studies similar to this, across the diversity of existing sesamoids, are necessary to uncover any fundamental principles which govern sesamoid development, both in evolutionary and ontogenetic contexts.

## Data accessibility

The full finite-element model used in this study can be downloaded from figshare (doi:10.6084/m9.figshare.4600309), as well as the original CT (doi:10.6084/m9.figshare.4600312) and MRI (doi:10.6084/m9.figshare.4600339) scans used in the construction of the model.
